# Evaluating a Website to Teach Children Safety with Dogs: A Randomized Controlled Trial

**DOI:** 10.3390/ijerph13121198

**Published:** 2016-12-02

**Authors:** David C. Schwebel, Peng Li, Leslie A. McClure, Joan Severson

**Affiliations:** 1 UAB Youth Safety Lab, Department of Psychology, College of Arts and Sciences, University of Alabama at Birmingham, Birmingham, AL 35294, USA; 2 Department of Biostatistics, School of Public Health, University of Alabama at Birmingham, Birmingham, AL 35294, USA; pli@uab.edu; 3 Department of Epidemiology & Biostatistics, Dornsife School of Public Health, Drexel University, Philadelphia, PA 19104, USA; lam439@drexel.edu; 4 Digital Artefacts, LLC, Iowa City, IA 52240, USA; joan@digitalartefacts.com

**Keywords:** dog bite, safety, cognitive development, website, ehealth, prevention

## Abstract

Dog bites represent a significant threat to child health. Theory-driven interventions scalable for broad dissemination are sparse. A website was developed to teach children dog safety via increased knowledge, improved cognitive skills in relevant domains, and increased perception of vulnerability to bites. A randomized controlled trial was conducted with 69 children aged 4–5 randomly assigned to use the dog safety website or a control transportation safety website for ~3 weeks. Assessment of dog safety knowledge and behavior plus skill in three relevant cognitive constructs (impulse control, noticing details, and perspective-taking) was conducted both at baseline and following website use. The dog safety website incorporated interactive games, instructional videos including testimonials, a motivational rewards system, and messaging to parents concerning child lessons. Our results showed that about two-thirds of the intervention sample was not adherent to website use at home, so both intent-to-treat and per-protocol analyses were conducted. Intent-to-treat analyses yielded mostly null results. Per-protocol analyses suggested children compliant to the intervention protocol scored higher on knowledge and recognition of safe behavior with dogs following the intervention compared to the control group. Adherent children also had improved scores post-intervention on the cognitive skill of noticing details compared to the control group. We concluded that young children’s immature cognition can lead to dog bites. Interactive eHealth training on websites shows potential to teach children relevant cognitive and safety skills to reduce risk. Compliance to website use is a challenge, and some relevant cognitive skills (e.g., noticing details) may be more amenable to computer-based training than others (e.g., impulse control).

## 1. Introduction

Pediatric dog bites are a significant public health problem. The Centers for Disease Control (CDC) estimate that about 4.5 million dog bites occur annually in the US [1]. Although dog bites affect people at all ages, children suffer the highest risk by a large margin [1,2]. The precise reason for elevated risk to children is unknown, but experts attribute it to at least three factors: (a) children behave in unpredictable and active ways [3], stressing dogs and sometimes causing animal aggression; (b) children lacking cognitive skills needed to recognize, understand, and behave appropriately near dogs; and (c) children, and especially younger children, are shorter than adults, leading to increased prevalence of bites to the head/neck region, which involves greater injury and more substantial treatment than bites to the limbs [4,5]. The present study developed and then evaluated a child-focused website to reduce pediatric dog bite risk by addressing the first two factors, children’s behavior near dogs and their cognitive skills that influence those behaviors.

Experts promote reduction of child dog bite risk through a range of strategies, including animal control, modification of environments, and increased adult supervision. We focused on another strategy recommended by experts [6,7,8], altering children’s risky and provocative behaviors near animals. Over the past few decades, public health experts have developed a number of programs with this goal in mind [6,7,8]. Existing programs implement a wide range of techniques to train children in safety with dogs, including classroom-based instruction with live dogs; video-based training in laboratories, classrooms, and hospital settings; and computer software programs [6,7,8]. Taken together, the intervention approaches tend to achieve some success; the most recent meta-analysis concluded that video-based interventions were successful at improving children’s knowledge but not changing their behavior in simulated or live interactions with dogs [8]. Most effective at changing children’s behavior were interventions that incorporated lessons on recognizing dog’s emotions and desires in the context of experiential interactions with live dogs [9,10].

To influence public health, behavioral interventions must be implemented and disseminated broadly. However, the most effective interventions to reduce child dog bite risk to date in this still-youthful and comparatively small literature with mixed scientific rigor—cognitive training incorporated into experiential interactions with live dogs—are financially and logistically difficult to disseminate broadly. An alternative that has been used successfully to teach children other injury prevention skills (e.g., pedestrian and bicycling safety [11,12]; fire safety [13]) is interactive computer-based training. With interactive computer-based training delivered via internet or mobile app, children can be engaged in rewarding and enjoyable activities that teach relevant cognitive skills and safety lessons. One such program, The Blue Dog, has been evaluated as a mechanism to teach children dog safety skills. Both children aged 3–6 and parents report The Blue Dog to be entertaining and engaging [14,15], and empirical testing suggests that children learn basic knowledge about safe engagement with dogs after using the program [14,15]. There is inadequate evidence currently, however, that the increase in children’s knowledge translates to safer behavior while engaging with dogs in simulated or live situations [15].

The present study adopted ideas from both lines of previous research. We implemented cognitive training through computer-based training programs, games and videos. We also introduced children to interactive computer-based training. Our goals were to improve children’s knowledge and behavior with dogs and also to provide opportunity to practice and improve cognitive skills relevant to safety with dogs. In doing so, we recognized the complexity of individual pediatric dog bite incidents. The complexity of a young child’s behavior, a dog’s behavior, and environmental context creates a challenging picture for interventionists to address. Undeniably, a wide range of factors contribute to children’s dog bites, but our analysis indicated three cognitive skills that seemed particularly relevant to children’s safety around dogs and comprised our primary focus of cognitive training: impulse control, perspective-taking, and noticing details. As detailed below, each of these skills develops rapidly between the ages of two and four, the age range when epidemiological data also indicate a peak in dog bite injuries [16].

Ability to control one’s impulses is critical to safe engagement with dogs, as impulsive play incorporating aggression, rapid or unexpected movements, and/or attempts to possess property dogs believe is theirs can surprise or upset dogs. Impulse control develops quickly during the preschool years [17] and although there are some developmental limitations concerning the extent to which inhibition can be taught, there is evidence aspects of impulse control can be taught and learned, including via interactive computer games [18].

The ability to take others’ perspectives is also still developing in early childhood. The literature focuses primarily on understanding the perspective of other humans, but ability to interpret a dog’s perspectives by cognitively processing signs of hunger, fatigue, or desire to play, may support safe engagement with dogs. Both classic Piagetian thought [19] and more contemporary theory of mind research [20] suggest that perspective-taking skill begins to emerge around age three and develops rapidly between the ages of three and five, reaching adult levels by middle childhood [21]. Perspective-taking skill also has been demonstrated to be trainable with practice [22,23], including via computer training [24].

Recognition of details requires development of finer aspects of visual perception, including contrast sensitivity, perceptual differentiation, and integration of the perceptual and cognitive systems. These skills develop rapidly through the preschool and early school years [25,26,27,28]. With those skills and concomitant development of perspective-taking skills, children may have better ability to recognize and then cognitively process subtle differences that help them determine a dog’s mood and desires.

To evaluate the efficacy of our intervention, we implemented a randomized controlled trial with 69 four- and five-year-old children. We conducted an assessment battery evaluating knowledge and safe behavior with dogs, plus relevant cognitive skills, both before and after children used the website at home for three weeks. A randomly-assigned control group was exposed to a transportation safety website over the same time period. We expected children exposed to the dog safety website would experience greater change in knowledge about dog safety, safe behaviors with dogs, and relevant cognitive skills (impulse control, perspective taking and noticing details) compared to the control group.

## 2. Methods

### 2.1. Participants

**Figure 1:**
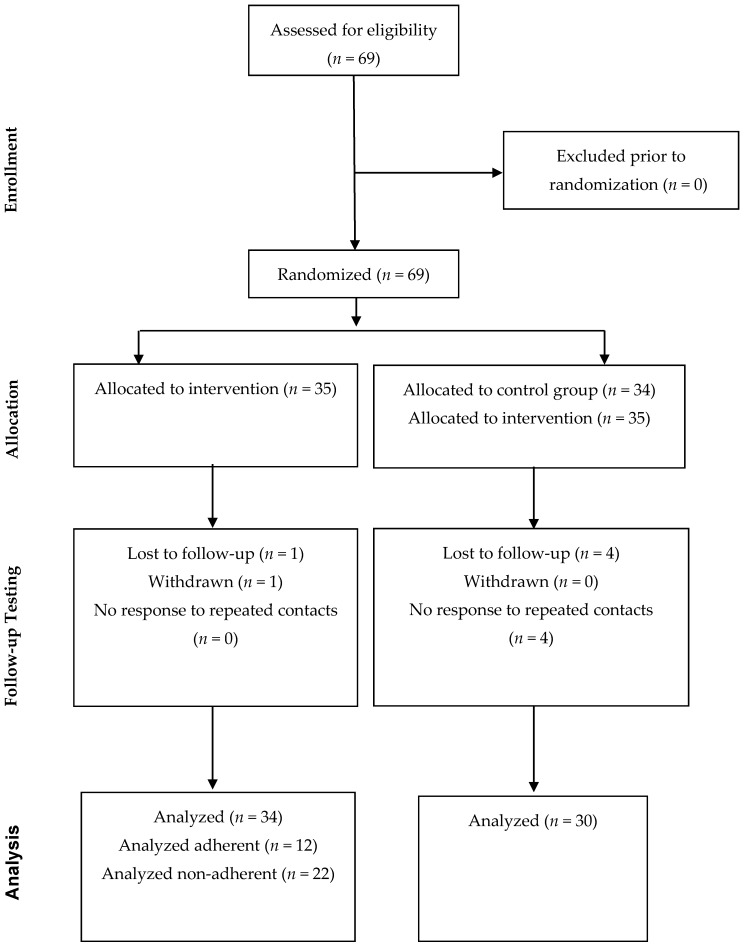
CONSORT flowchart of study enrollment.

Sixty-nine 4- and 5-year-old children were recruited from a laboratory database of local families interested in participating in research in the Birmingham, Alabama area and randomly assigned to one of 2 training conditions, exposure to the intervention website on dog safety or the control website on transportation safety. Database families are derived from a number of community sources, including contact at local schools, word-of-mouth, and flyers in community settings such as libraries and supermarkets. See Figure 1 for a CONSORT flowchart of participation and attrition; of the 69 children randomized to condition, 64 completed the study (93%). Inclusion criteria included: children in correct age range (4–5 years); children regularly exposed to pet dogs at home or at the home of friends, neighbors, or relatives; and family accessibility to reliable internet access. Exclusion criteria were minimal: inability to speak (child) or read (parent) in English or a disability that prohibited valid participation in the study protocol. No families were excluded for these reasons. The study was exempt from registration as a clinical trial due to being a behavioral trial in the early phases of testing.

The randomized sample of 69 children was 48% male and had an average age of 5.1 years (standard deviation (SD) = 0.59, range = 4.00–5.98). It was racially diverse, with 67% of parents identifying their children as White, 25% as African American, and 9% as other races/ethnicities, or as bi-racial or multi-racial. All participants’ parents provided written informed consent, and children provided verbal assent. The study was conducted in accordance with the Declaration of Helsinki, and the protocol was approved by the Institutional Review Board at the University of Alabama at Birmingham (Protocol X130802002). Both children and parents were free to request withdrawal from the study at any point.

### 2.2. General Protocol

Families completed two laboratory visits that sandwiched use of the randomly-assigned website at home (average website usage window = 16.93 days, SD = 5.45; details about websites appear in Section 2.3 and Section 2.4). During each visit to the university laboratory, children engaged in a number of assessments and parents completed written questionnaires; these sessions were each a single visit for about 90 min and details appear below. Website usage at home was permitted on desktop or laptop computers. Parents were sent emails within 24 h/next business day after the initial visit with instructions on how to access their child’s assigned training website. Parents then received reminder emails every fourth business day prior to the post-intervention visit to encourage use of the assigned website. Technical troubleshooting was provided if needed.

### 2.3. The Dog Safety Website

An overarching goal of the website design was to make it both educational and entertaining. We sought to promote frequent website use via game-like components that also taught children basic skills in safety with dogs as well as encouraging practice and learning of relevant cognitive skills. The website was designed with two primary screens for children, games and videos. All website material was developed in consultation with veterinarians, dog behavior experts, child psychologists, public health specialists, and parents and children. Table 1 offers an overview of the child-oriented components of the website.

The games screen offered eight interactive and entertaining games for children to engage in, none of which required literacy (see Table 1). Previous research suggests that children readily learn cognitive, health and academic skills from interactive educational computer games [18,24,29]. One of the eight games was designed to teach basic knowledge about safety with both familiar and unfamiliar dogs. Presented like a trivia contest, children heard and saw written language presenting questions and then selected the answer they believed to be correct, with oration occurring to “read” aloud any text where the mouse was laid. Sample question topics included responding to a dog that is barking at you and identifying appropriate activities to engage in with a dog. Feedback was provided to ensure that children learned the correct answer and the reason why before moving on to the subsequent question. The remaining games were designed to teach one or more of the target cognitive skills of impulse control, perspective taking and noticing detail. Some were variants of “classic” neuropsychological and developmental tasks.

For example, a Stroop-like task to teach impulse control involved responding to pictures of carrots, some of which were green and others purple. Similarly, perspective taking was practiced through “classic” theory of mind scenarios like false belief tasks presented in animated computer dog-themed games. Other games were more novel. One perspective-taking game, for example, asked children to distinguish “dog” toys like bones and chew-toys from “child toys” like a toy truck. A task to practice noticing of details asked children to identify differences in a series of two similar drawings. All games were designed to be engaging, colorful, and rewarding to maintain children’s interest and return visits.

The videos screen offered five testimonial videos and eight videos in a series entitled “through the dog’s eyes”, all of which were scripted and created for the website. The testimonial videos consisted of multi-racial child actors/actresses describing incidents when they had been bitten by a dog and the lessons they learned from those bites. As outlined in Table 1, basic information about dog safety was conveyed in emotional stories designed to increase viewer’s perceived vulnerability to bites. Animation was integrated into the short film clips to show bite incidents. A substantial body of research supports the use of testimonials to create health behavior change [30,31].

The “through the dog’s eyes” videos were designed primarily to teach perspective taking and also incorporated lessons on safety with dogs and noticing details. Each short video was filmed from the dog’s perspective as the dog negotiated scenes like parks, sidewalks, and living rooms. A narrator told what the dog was seeing and much of the filming was conducted with a camera attached to dogs’ collars.

The website incorporated one other key component to maximize effectiveness, interest, and engagement: a point and level system whereby children advanced through levels of accomplishment. Only some games and videos were available at the start, and as children played those games and viewed those videos, they earned points that advanced them to the next “level”. Each level was marked by a breed of dog, and advancement was accompanied by a printable certificate of achievement. With each level of advancement, children “unlocked” new games and videos to engage with. This form of behavioral reinforcement is widely cited as effective in health behavior change [32]. We expected all children to progress through each of the 10 levels.

The use of a point and level system also enabled us to educate and inform parents about child safety with dogs. Each time children achieved a new level (after about 15 min of engagement on the website), a message was sent to parents via email to congratulate the child and offer parents the opportunity to congratulate. Equally important, the message included targeted information about the children’s learning, allowing parents to reinforce the message at home. Reaching busy parents to deliver safety messages is challenging. Delivery of brief congratulatory but also educational messages via electronic means permits parents to be informed about safety topics without substantial time or effort.

The website is not currently available to the public but the authors of this manuscript are happy to discuss cooperation with interested and qualified scholars to further study or extend aspects of the website.

### 2.4. The Transportation Safety Website: Control Condition

Children randomly assigned to the control condition used the “Otto the Auto” website [33], which was publicly available at the time of the study (March 2015–January 2016) but apparently is no longer accessible to the public. Like the dog safety website, Otto the Auto involves several interactive games, activities and stories. It is developmentally appropriate for this age group and teaches a range of skills and lessons on safety while walking, bicycling, and driving.

### 2.5. Pre- and Post-Intervention Assessment

Laboratory pre- and post-intervention assessments lasted about 1.5 h and were very similar (minor differences noted below). Children completed a battery of tasks, detailed below. Full instruments and the study protocol are available from the authors upon request from qualified and interested scholars.

#### 2.5.1. Assessment of Knowledge/Behavior with Dogs

Four tasks assessed children’s knowledge and behavior with dogs. First, children completed a nine-item “quiz” on safe behavior with dogs. Sample questions are, “Is it OK to play rough with a dog if it seems to enjoy it?” and, “Is it OK to pet a dog gently when it is sleeping?”. Experimenters read the questions orally and children answered “yes” or “no”. The number of correct responses was summed, with higher scores indicating greater knowledge. Second, children looked at a series of 16 photos, some of which showed dogs that were safe to play with and others of which showed dogs that would not be safe to play with (e.g., in a crate, eating) [15]. The number of correct responses to the dangerous situations was summed, with higher scores indicating greater recognition of safe behavior with dogs. Third, children engaged in 7 simulated scenarios using a dollhouse that included child and dog characters, realistic furniture, and a yard [15]. In each scenario, children heard a brief scene and then explained and used the dolls to act out what would happen next. For example, the experimenter would use the dolls to act out a child playing in the kitchen near dog food and then the doll dog entered, saw the child, and approached the food bowl. The experimenter said, “[Child’s Name] is playing around in the kitchen near [Dog name’s] food. [Dog’s name] comes into the kitchen and sees [Child’s Name] near his/her food bowl making him/her upset and start to growl. What will happen next?” The task was videotaped and then coded using objective coding criteria to score the child’s response as safe (1 point), safe but not optimal (0.5 points), or unsafe (0 points). Scores across the seven scenarios were summed to yield a single score of recalling dog safety in the dollhouse task, with higher scores indicating better knowledge about safety. Inter-rater reliability was established on 30% of the sample between two independent coders before coding videotapes and was strong (kappa = 0.90); data from the primary coder was used for analysis.

Fourth, children engaged with a live dog in a protocol that included both unstructured and semi-structured activities (adapted from [15]). This assessment occurred only during the post-intervention visit as previous research raised concern that repeated exposure to an unfamiliar dog in a laboratory setting may result in biased, more comfortable or risk-taking behavior on the second occasion [15]. During the live dog protocol, children entered a large room that contained a research assistant and dog handler seated in a corner of the room, an unleashed therapy dog, a dog bed, three small “dog” toys, and three mundane child toys. The protocol involved four stages. In the first stage, children were introduced to the dog and handler and were told that the adults (the researcher and handler) had “work” to do so the child should do whatever he/she would like. The child and dog toys were routinely identified. During this time period, both the handler and the researcher remained alert to activities in the room and intervened if they sensed concern about the safety of the child or the dog. No such instances arose. After three minutes, children were told they could play with the dog and asked to select from one of three activities: throwing a ball for the dog to retrieve, brushing the dog’s fur, or feeding the dog a treat (stage 2). This activity was monitored by the adults in the room for safety, but no interventions were required. In the third stage, children were told the dog needed to rest and instructed to do whatever they wanted while the dog rested and the adults worked more. This stage lasted 3 min. In the fourth and final stage, children were told that they could play with whatever they wanted in the room, including the dog and the other activities they had not chosen in stage 2. The researcher and handler stayed alert and in the room for the final two stages of the protocol also to protect the safety of both the child and the dog, but had no cause to intervene during any of the sessions. Children and parents were debriefed about safety with dogs following the research session.

Children’s behavior during the live dog interaction was videotaped through a one-way mirror and then coded using objective criteria. Factor analysis of the coding yielded a one-factor solution with seven measures as the best way to describe patterns in the data; those seven measures were then standardized and averaged to create a composite of the child’s risk-taking with the live dog (average intercorrelation = 0.50; Cronbach’s α = 0.65). These criteria included measures of when and how the child touched the dog, the extent to which the child was close/intimate with the dog, whether the child handled the dog’s toys, and whether the child interrupted the dog during its “rest time”. Higher scores indicated greater risk-taking. Inter-rater reliability for coding was obtained on 24% of the sample and was adequate (kappa = 0.84 for categorical variables and correlation >0.99 for continuous variables such as time before first contact with the dog).

#### 2.5.2. Assessment of Perspective Taking

We assessed perspective-taking with three tasks. First, children completed a standard first-order false belief task (the “Smarties” task [34]). Children were exposed to a package that had labeling and appeared to contain one set of contents but actually contained something else inside. In the pre-lab intervention, children were exposed to a candy box containing pencils. Because children had been exposed to that task in the first visit, the post-intervention lab used a bubble gum package with an eraser inside. In both cases, children were exposed to the original wrapper and then allowed to open it, revealing the unexpected contents. After those exposures and time to process what they had seen, children were asked what they thought a grown-up who was not in the room would think was inside the package. A single dichotomous score resulted (correct answer of what appeared to be inside vs incorrect answer of what was actually inside).

Second, children completed standard appearance-reality tasks where they were asked to distinguish the appearance and reality of eight objects, four of them with ambiguous appearances (sponge that looked like a rock, magnet that looked like candy, a rubber ice cream cone, and a candle that looked like an apple) and four without ambiguity (spoon, mug, plate, toothbrush) [35]. After an introductory training period, the researcher individually presented each object to the child and asked, “Is this something that looks the way it really and truly is?”. Children were required to answer YES or NO to the question and the total number of correct answers out of 8 was summed.

Last, children completed the Test of Emotion Comprehension (TEC; [36]), a battery of tasks designed to assess children’s ability to comprehend emotions. We focused especially on the children’s responses to Component I (Recognition), which consists of the correct number of answers to five items asking children to recognize the emotion identified among a set of four drawings; Component II (External Cause), which consists of the correct number of answers to children’s responses across five items in which he/she is asked to recognize the emotion inferred by an emotion-laden situation the character faces among a set of drawings; and the overall score, which is derived as a pass/fail score across all nine components of comprehending emotions (possible range = 0–9). The TEC has strong psychometric properties [37,38]. The five measures of perspective taking were modestly correlated (average intercorrelation = 0.41) and were standardized and then averaged into a single construct, with higher scores reflecting greater perspective-taking ability.

#### 2.5.3. Assessment of Noticing Details

We assessed children’s ability to notice details using two measures. First, children completed three subtests of the Test of Visual Perceptual Skills—3rd Edition (TVPS-3; [39]), Visual Discrimination (in which the child points to the design that matches a target design among several choices), Spatial Relationships (in which the child chooses the design that differs from the rest among a series), and Visual Figure Ground (in which the child identifies one design among many within a complex background). The TVPS has adequate psychometric properties (Cronbach’s alphas for three subtests of interest are 0.76, 0.87, and 0.82; [39]). Scores are reported as scaled scores (Mean = 10, SD = 3). Second, children completed an embedded figures task in which they were asked to locate 13 figures embedded in a complex black-and-white drawing [40]. Children circled identified figures with a colored pencil; the total number identified within 4 min was tallied, with higher scores representing greater skill in visual perception of details.

The four measures of noticing details were modestly correlated (average intercorrelation = 0.28) and were standardized and then averaged into a single construct, with higher scores reflecting greater ability to notice details.

#### 2.5.4. Assessment of Inhibitory Control

Inhibitory control was assessed using a battery of five laboratory-based assessment strategies adopted from previous research [17,41]. First, children played the “Simon Says” game, in which they viewed a short video of a model leading them to engage in simple behaviors like touching their head or clapping their hands if “Simon Says” to do so, but were supposed to inhibit engaging in the behavior if “Simon doesn’t Say to do it”. Simon Says was implemented in two short sessions, each with 18 tasks; nine required inhibition. The total number of tasks in which children correctly inhibited was summed. Second, children engaged in “Walk a Line”. In this task, children walked along a 3.2 m line three times, first at “typical” speed, second as “fast as you can”, and third “as slow as you can”. The walks were recorded and then timed. Inhibition was measured as the difference between the “typical” and “slow” walking speeds, with greater differences reflecting better inhibition. Third, children engaged in “Draw a Circle”. Similar to the line-walking task, children were presented two concentric circles on a sheet of paper and asked to draw a new circle between the two on the page. Three sheets were presented and children drew the circles at “typical” speed followed by “as fast as you can” and “as slow as you can”. The difference between the typical drawing and the slow drawing was computed as a measure of inhibition.

Fourth, children completed the “Long Speech” task. Children listened to the researcher read a 90-second descriptive passage from John Steinbeck’s *The Grapes of Wrath* [42]. A dichotomous score was tallied if the child interrupted the researcher while reading the passage (scored 1 for lower inhibition) or if the child did not interrupt (scored 2). Last, children engaged in the “Prize Bin” task in which they were presented a large bin with a wide range of toys at the end of the experiment and told to look carefully through the options and choose two toys to take home. The amount of time children took to select their two prizes was recorded as a measure of inhibition. The five measures of inhibitory control were modestly correlated (average intercorrelation = 0.25) and were standardized and then averaged into a single construct, with higher scores reflecting greater inhibition.

### 2.6. Other Measures

Basic demographic information such as child age, parent gender, and family socioeconomic status was reported by parents at the initial laboratory visit. During the post-intervention laboratory visit, children and parents independently completed brief surveys concerning their impressions and enjoyment of the website.

### 2.7. Analysis Plan

Data analysis proceeded in six steps, all using SAS 9.4 (Cary, NC, USA). Descriptive data were considered first, both across the full sample and within randomly-assigned groups. Differences across intervention groups were evaluated. Second, we examined adherence to the intervention protocol, defined as completing all ten levels of achievement or coming close to it (completing nine of ten levels). Because adherence was poor among a portion of the sample, three groups were formed: children randomly assigned to the transportation safety website (control), children randomly assigned to the dog safety website who were adherent to using the website, and children randomly assigned to the dog safety website who were not adherent to the website. Descriptive data for the three groups plus the overall sample were considered.

Third, in an intent-to-treat analysis, our primary hypothesis was tested using general linear regression models (GLM) on the following outcome measures: dog knowledge “quiz”, recognition of safe behavior in dog photos, recall of safe behaviors in the dollhouse simulation, engagement of safe behaviors in the interaction with the live dog, and cognitive processing via aggregated constructs assessing perspective-taking, noticing details, and impulse control. Fourth, given poor adherence to the intervention, we re-computed the GLM models in a per-protocol analysis which compared the control group (transportation safety) to the children who were compliant to the intervention in the dog safety group. Fifth, we computed GLM models comparing all three groups (transportation safety control group, compliant dog safety group, and non-compliant dog safety group) for all outcome measures. Finally, we considered descriptive reports from the sample on their impressions of the websites, including the extent to which they enjoyed using them.

## 3. Results

Table 2 lists demographic data of the sample, both overall and by randomly-assigned intervention group. Statistical tests of differences between the groups yielded a significant result for gender; despite randomization, the transportation safety group was 61.8% male and the dog safety group was 34.3% male. Thus, gender was controlled for in subsequent analyses. No other demographic variables were different across groups.

We next examined adherence to the dog safety intervention. As shown in Table 3, 12 children (36%; *n* = 10 who achieved all ten levels of the website over the intervention period and *n* = 2 who reached level 9) were considered compliant to the intervention. None of the remaining children surpassed Level 5 and 30% reached only Level 1 or 2. Some of that subsample of children was exposed to a small amount of training (those at Level 1 likely did nothing beyond signing in), but in most cases a very small amount, and therefore this group was considered non-compliant for subsequent analyses. Regression models with compliance as an outcome and all available demographic variables as predictors yielded no significant differences between compliant and non-compliant children on demographic characteristics. We did not consider use or non-use of the control transportation safety website a major influence on interpretation of the study’s results and left that group coherent as a single group for analysis.

Table 4 presents descriptive data on the primary outcome variables for the control group (*n* = 30), the compliant portion of the intervention group (*n* = 12), and the non-compliant portion of the intervention group (*n* = 22). Table 5 presents the intent-to-treat analysis testing our a priori hypothesis that exposure to the dog safety website would yield safer behavior and improved cognition compared to exposure to the control transportation safety website. As shown, just one statistically significant result emerged: children exposed to the dog safety website had significant improvement in their ability to notice details compared to children in the control group exposed to the transportation safety website. Table 6 presents the per-protocol analysis comparing children who were compliant to the dog safety website intervention versus those in the transportation safety control group. Three statistically significant results emerged. Specifically, children exposed to the dog safety website demonstrated significant knowledge about (quiz) and recognition (dog photos) of safe behavior with dogs, and they showed a significant increase in their ability to notice details compared to children in the control group.

Table 7 presents the GLM analysis comparing changes between pre and post scores across the three groups, with age, gender and pre scores controlled for (except the model for the live dog behavior, which used post scores as the outcome and did not control for pre scores since they were not collected). Post-hoc testing replicated the per-protocol analysis and yielded differences between the compliant children in the dog safety group versus the control transportation safety group in the knowledge quiz, dog photos, and noticing details composite outcomes.

The final step of our analysis was to consider descriptive data on parent and child perceptions of the website, including their enjoyment of it, following the intervention period. As shown in Table 8, children who were compliant to the dog safety intervention rated the website as enjoyable and tended to believe other children would enjoy using it. Parents of children compliant to the dog website intervention rated their children’s enjoyment as very high and tended to believe they would continue using the website in their home as well as recommending it to other families.

## 4. Discussion

Each individual pediatric dog bite incident has a complex set of etiological factors. These include behaviors of the child and the dog as well as environmental context. Children’s immature cognitive skills often play a role in dog bites, however, as they lead young children to behave in ways that may provoke dogs to bite them. In this study, we developed and then evaluated an interactive website to teach children rules about safety near dogs, learn and hone cognitive skills required to make safer decisions near dogs, and gain exposure to website content that might increase their perceived vulnerability to bites. Results indicate that children who were compliant to using the website at home for about three weeks achieved better knowledge about safety with dogs and were better able to recognize safe behaviors with dog in photos. They also showed improvement in their cognitive skill to notice details after exposure to the website and reported that they enjoyed using the website. Other outcome measures did not yield statistically significant results, nor did intent-to-treat analyses that included non-compliant children in the active intervention group.

The result that our experiment yielded improved recognition about safe dog behavior is encouraging and replicates previous results evaluating The Blue Dog software [14,15]. Together, the findings suggest that children can learn basic knowledge about how to behave more safely around dogs through training in an interactive computer-based format that is easy to administer and disseminate. The fact that we achieved improvement in children’s ability to notice details is also encouraging. We worked with children who were at the age when we anticipated they could learn those skills (in classical terms, Vygotsky’s “zone of proximal development” [43]) and apparently the practice compliant children received while engaging on the website was sufficient to learn some of those relevant cognitive skills.

Despite these positive outcomes, however, we also discovered several null results. Most prominent perhaps, we failed to detect any change in children’s simulated or actual behaviors with dogs following the intervention. Interpreting null results must be conducted with utmost caution, but given the under-developed state of this literature we believe it worthwhile to speculate about possible reasons for the null results we obtained. Ocular analysis of descriptive data in Table 4 suggests that children’s scores in the dollhouse task increased over time in all three groups, but that the change in the children compliant to the dog safety intervention achieved slightly larger increases than children in the other groups. It may be that children receiving any safety-related intervention, including those that completed only minimal training with the active intervention (non-compliant dog safety group) and those who may have generalized lessons from other safety-based training (transportation safety group) learned some lessons relevant to behaving more safely in the simulated stories. Children who received the desired training in the target domain of interest (compliant dog safety group) yielded the most change, but that change was not significantly different from what was witnessed in the other groups.

The live dog protocol, which involved exposure to an unfamiliar therapy dog using a mix of unstructured and semi-structured interactions, also yielded null results. Although the live dog protocol offered the advantage of a strategy to assess children’s behavior with an actual dog, it suffered from the limitations of children encountering an unfamiliar dog in the somewhat artificial situation of a university laboratory; using a range of different dog sizes, breeds, and temperaments across children; and creating a somewhat artificial interaction between dog and child with unfamiliar adults in the room that does not accurately represent a high-risk home environment with a familiar pet dog. We also conducted only post-assessment measure for that measure, as our previous research suggested that the use of this measure on two occasions yielded different and potentially invalidating behavior on the second occasion [15]. Despite these limitations, the non-significant trend in descriptive data (Table 4) was as hypothesized, with compliant children in the dog intervention group showing the least risk-taking, non-compliant children in the dog intervention group falling in the middle, and children in the control transportation safety group taking the greatest risks with the dog.

We also discovered null results for two cognitive skill constructs, impulse control and perspective taking. Efforts to improve aspects of cognitive skills through interactive computer training are well-documented in the scientific literature [44], including among children [45]. We speculate the reasons for null results concerning impulse control and perspective-taking may be different. Despite some positive evidence [18], impulse control is difficult to teach in any age group, including via computer-based practice and especially among young children [46,47]. Innate biological factors likely contribute significantly to impulsive and disinhibited behavior during the preschool years [48] and behavior patterns may be resistant to change efforts. Unlike impulse control, perspective-taking has been taught successfully on multiple occasions to children as young as 3 and via computer-based training programs [49]. In this case, our computer-based teaching strategies may have been insufficiently interactive to children’s responses, may not have offered sufficient practice to encourage change, or may not transfer across contexts from computer-based training to standard lab tasks like theory of mind and the TEC. Future efforts to teach children perspective-taking skills relevant to safety with dogs might consider techniques that will deliver training more clearly from the context of understanding a dog’s desires and reconciling those desires with what the child would prefer to do (e.g., child wants to play but dog wants to sleep).

The fact that the per-protocol analysis yielded some significant results but our intent-to-treat analysis did not amplify a message that most behavioral interventionists recognize: prevention programming only works if the target audience adheres to the intervention protocol. Our website was designed to be interactive, engaging, and entertaining, and was rated as entertaining by children who used it. It incorporated a behavioral reward system such that children were rewarded for frequent play and motivated to return to the website frequently. It also incorporated messaging to parents, who we presumed would reinforce the lessons children learned and encourage continued engagement with the website. Despite these components, nearly two thirds of the children randomly assigned to use the dog safety website were non-compliant with website usage. Anecdotally, families attributed non-compliance to a range of reasons including busy schedules, computer/technical problems, and forgetfulness. Solutions to the adherence challenge are difficult. One option is to mandate usage, for example by incorporating the training into a preschool curriculum or community center programming. This solution suffers from the limitations that administrators may be reluctant to squeeze dog safety training into already overburdened curricula or programming and that the intervention may not reach all children in the target age group. Another option is to continue to search for innovative ways to create public health websites for children that are highly engaging, entertaining, and motivating. This solution is promising, but will require interdisciplinary creativity and innovation to extend existing efforts. A third option is to assume that a portion of the population will be non-adherent but promote usage as widely as possible to extend reach to those willing to accept it. This solution suffers from the reality that distribution may be less than desired.

This study had several strengths, including use of a randomized controlled design and multiple outcome measures to evaluate the hypotheses. It also had limitations. It is difficult to measure children’s risk-taking with dogs in a valid and ethical manner. We coped through use of a live dog interaction, simulations using a dollhouse, and more “standard” knowledge-based questioning and identification of risks in photographs, but each of those measures suffers from methodological flaws. We used a short-term follow-up assessment only and did not assess long-term retention of lessons learned. We also were limited by our sample size, especially for the per-protocol analysis given the non-adherence among many participants. The sample of 46 used in that analysis had power =0.31 to detect a medium effect size. Future work should address noncompliance and recruit larger sample sizes to have sufficient power to detect differences. Our sample included moderate diversity in household income, parental education, and child race/ethnicity, but it was on average wealthier, better educated, and more white than the local or national population, so generalization of results should be conducted cautiously. Finally, our protocol included lengthy assessment periods of rather young children. Although we noted no evidence of fatigue, inattention or boredom, and we implemented an interactive study protocol with frequent breaks, it is possible that the lengthy assessment influenced results.

Future work might also consider other aspects of the child–dog interaction that could lead to biting situations. We sought to teach basic cognitive skills and assumed they might generalize to a wide range of potentially-risky situations with dogs. We did not specifically evaluate whether this assumption of causality was correct, but post-hoc analyses did find significant correlations between some composites of cognitive function and dog safety outcomes among the full sample. More broadly, we acknowledge the complexities of intervening to reduce child injury incidents through domain-specific training because child injury situations occur under a wide range of environmental contexts. Researchers should continue to consider how to develop interventions, including interactive websites with multiple educational components, so that the intervention appropriately generalizes to the wide range of injury-risk situations children might face.

It would be valuable also for future research to continue to consider the best ways to train children to handle the complex interactions they face with dogs. Animal control, modification of environments, and increased adult supervision will always play a role in pediatric dog bite prevention, but ultimately children’s behavior and decisions must also be addressed. The existing literature on dog bite prevention remains small in comparison to some other domains of child injury prevention, but suggests thus far that cognitive training, perhaps with some experiential or simulated interaction with live dogs, may be the most effective training strategy to date [6,7,8]. Other strategies should be considered, especially as simulation and virtual reality technology increases and may permit children to “experience” interactions with dogs without the ethical risks of interacting with live dogs or the potential confusion arising from interactions with trained therapy dogs that do not behave as a typical family or unfamiliar dog might.

Finally, future research should continue to consider the role of parents and other adult supervisors on children’s safety with dogs. We know that adult supervision—especially from parents but also from babysitters, nannies, grandparents, and so on—is extremely valuable to reduce child injury risk [50,51]. Our website used brief and succinct messages to parents because we recognize contemporary parents are bombarded by many demands on their time and we reasoned quick email messages might be read and processed more effectively than lengthy written or video messages. This reasoning might be re-considered and evaluated in future research, as education of parents as well as children will be critical to achieve greater success in reducing pediatric dog bite injuries.

## 5. Conclusions

In summary, our results offer initial promise for an interactive and entertaining website to train children in safety with dogs via teaching of basic rules about safety, cognitive training in relevant skills, video-based learning including testimonials to alter perceived vulnerability to risk and change behavior norms, and messaging to parents who could reinforce children’s online lessons. Among the group of children who were compliant with using the website regularly at home, we saw significant change in children’s knowledge and recognition of safe behavior with dogs as well as one relevant cognitive construct, the ability to notice details. The children reported that they enjoyed the website. Data trends were generally in the hypothesized direction for other variables of interest, but were not statistically significant. A significant limitation to our study was the lack of compliance among a large portion of the sample. Next steps for the field might include identifying ways to improve compliance with use of an educational website on dog safety, refinement of the website, and then implementation of a larger randomized trial. If results from a larger trial extend these initial results, and especially if they yield behavior change, website release to the public and broad dissemination through partnership with industry, non-profit entities, and government bodies could be explored.

## Figures and Tables

**Table 1:** Components of the Dog Safety Website.

**Games: Interactive Activities for Cognitive Learning**		**Lesson and Source**
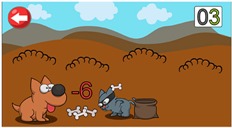	Bones	Inhibition, adapted from Iowa Gambling Task
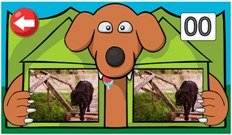	Spot the Difference	Notice details, adapted from puzzles in popular children’s magazines
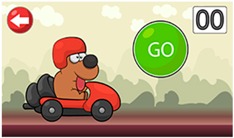	Driving	Inhibition and executive function, adapted from Go-No Go Task
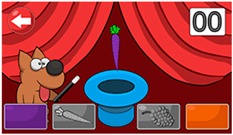	Magic	Inhibition, adapted from Stroop-like tasks
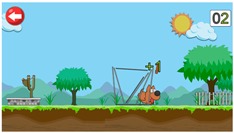	Slingshot	Inhibition, adapted from games like Angry Birds (Rovio Entertainment, Espoo, Finland)
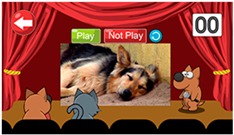	Cinema	Notice details, distinguish short scenes when it is safe or not safe to play with a dog
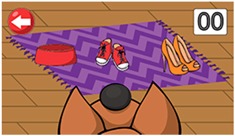	Perspectives	Perspective-taking, distinguishing child vs. dog items
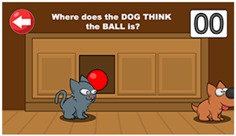	Ball Game	Perspective-taking, based on classic theory of mind false belief tasks
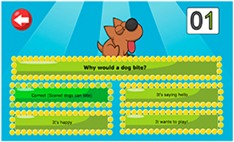	Trivia	Basic trivia and knowledge about dog safety
**Testimonial Videos: Learning the Lessons of Other Children**		**Lesson and Source**
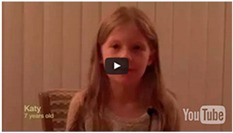	Bethany’s Story	Learning not to play with dogs that are eating
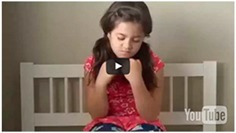	Emma’s Story	Learning not to play with dogs that are sleeping
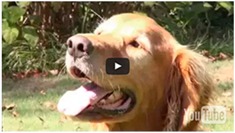	Katy’s Story	Learning not to fight with dogs over property
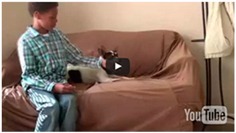	Morgan’s Story	3 lessons: (a) vulnerability to bites, (b) greeting dogs/perspective-taking, and (c) noticing details
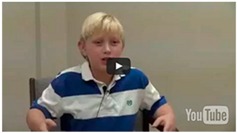	Thomas’ Story	2 lessons: (a) greeting unfamiliar dogs, and (b) petting dogs on the face
**“Through the Dog’s Eyes” Videos: Learning from the Dog’s Perspective**		**Lesson and Source**
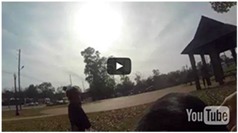	How I Like to Have Fun	Learning to recognize when a dog wants to play
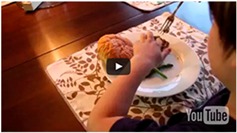	How I Like to Play Fetch	Knowing when to play vs. leave dog alone
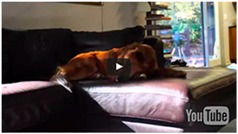	How to Know When I’m Feeling Sick	Knowing to leave sick dogs alone
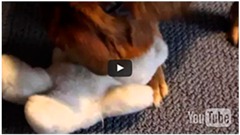	How to Know When I’m Ready to Play	Learning not to play aggressively/boisterously with dogs
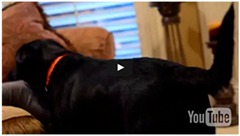	What to Do When I Take Your Toy	Learning not to fight over toys with dogs
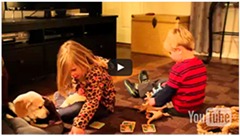	What to Do When I’m Asleep	Knowing when to play vs. let dog sleep
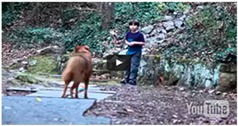	What to Do When I’m Eating	Knowing when to play vs. let dog eat
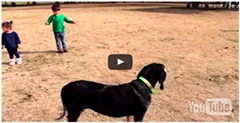	What to Do When We Meet	Knowing how to greet unfamiliar dogs

**Table 2:** Descriptive statistics: Mean ± standard deviation (SD) or *n* (%) of demographical characteristics of all participants randomized to condition.

Variables	Transportation Safety Group (*n* = 34)	Dog Safety Group (*n* = 35)	*p*-Value
Child’s age (years) ^a^	5.1 ± 0.6	5.0 ± 0.6	0.344
Mother’s age (years) ^a^	35.4 ± 5.1	37.0 ± 5.3	0.202
Father’s age (years) ^a^	39.1 ± 7.0	39.1 ± 6.2	0.986
Gender (% male) ^b^	21 (61.8%)	12 (34.3%)	0.022
**Ethnicity ^c^**			0.256
African American	10 (30.3%)	6 (17.1%)	
Caucasian	19 (57.6%)	27 (77.1%)	
Other	4 (12.1%)	2 (5.7%)	
**Mother’s education ^c^**			0.090
High School Diploma or less	2 (5.9%)	0 (0.0%)	
Some college/Associate’s Degree	10 (29.4%)	4 (11.4%)	
Bachelor’s Degree	9 (26.5%)	15 (42.9%)	
Post-Graduate/Graduate Degree	13 (38.2%)	16 (45.7%)	
**Father’s education ^c^**			0.786
High School Diploma or less	5 (15.6%)	3 (8.6%)	
Some college/Associate’s Degree	5 (15.6%)	7 (20.0%)	
Bachelor’s Degree	9 (28.1%)	12 (34.3%)	
Post-Graduate/Graduate Degree	13 (40.6%)	13 (37.1%)	
**Family Income ^c^**			0.357
Below 40,000 USD	8 (25.8%)	3 (8.8%)	
40,000–59,000 USD	6 (19.4%)	6 (17.7%)	
60,000–79,000 USD	2 (6.5%)	2 (5.9%)	
80,000–99,000 USD	5 (16.1%)	5 (14.7%)	
Above 100,000 USD	10 (32.3%)	18 (52.9%)	
**Number of adults in home ^c^**			0.280
1	9 (26.5%)	5 (14.7%)	
2	22 (64.7%)	28 (82.4%)	
3	3 (8.8%)	1 (2.9%)	
**Number of children in home ^b^**			0.730
1	5 (14.7%)	7 (20.6%)	
2	20 (58.8%)	17 (50.0%)	
3 or more	9 (26.5%)	10 (29.4%)	

^a^ Two sample *t* test; ^b^ Chi-Square test; ^c^ Fisher’s exact test.

**Table 3:** Adherence to dog safety website intervention: Levels achieved.

Level Achieved	Frequency
Level 1: Chihuahua	8 (24.2%)
Level 2: Dachshund	2 (6.1%)
Level 3: Pug	5 (15.2%)
Level 4: Basset Hound	3 (9.1%)
Level 5: Doberman	3 (9.1%)
Level 6: Poodle	0 (0.0%)
Level 7: Husky	0 (0.0%)
Level 8: German Shepherd	0 (0.0%)
Level 9: Boxer	2 (6.1%)
Level 10: Great Dane	10 (30.3%)

**Table 4:** Baseline and post measures across groups: Descriptive data. All values except those for “False belief” and “Long speech interrupt” are expressed as mean ± SD.

	Transportation Safety Group (*n* = 30)		Dog Safety Group: Compliant (*n* = 12)		Dog Safety Group: Non-Compliant (*n* = 22)	
Variables	Pre-IV	Post-IV	Pre-IV	Post-IV	Pre-IV	Post-IV
**Dog Knowledge/Behavior**						
Knowledge quiz (items correct out of 9 total)	5.5 ± 1.4	5.3 ± 1.7	6.0 ± 1.7	6.5 ± 1.3	5.4 ± 1.0	5.4 ± 1.8
Dog photos (correct of 8)	4.6 ± 2.5	5.1 ± 2.3	5.7 ± 1.7	6.7 ± 1.2	5.6 ± 2.4	5.4 ± 2.6
Dollhouse safe behavior (range 0–7)	3.0 ± 1.9	3.6 ± 2.1	2.1 ± 2.0	3.0 ± 2.0	3.5 ± 1.7	3.9 ± 1.8
Live dog risky behavior (average z-score)	NA	0.1 ± 0.8	NA	−0.2 ± 0.5	NA	−0.2 ± 0.6
**Perspective Taking Composite**	−0.6 ± 2.9	−0.2 ± 3.0	1.1 ± 2.9	1.0 ± 1.7	0.4 ± 3.3	0.6 ± 2.4
Appearance reality (correct of 8)	6.6 ± 1.3	6.4 ± 0.8	6.6 ± 1.8	6.4 ± 0.9	6.5 ± 1.6	6.0 ± 1.6
False belief (1/0)	27/7	24/6	10/2	9/3	20/3	20/2
TEC Component 1 (correct of 5)	4.5 ± 0.7	4.8 ± 0.6	4.9 ± 0.3	5.0 ± 0.0	4.6 ± 1.0	4.7 ± 1.1
TEC Component 2 (correct of 5)	4.0 ± 1.1	3.8 ± 1.2	4.3 ± 0.9	4.4 ± 0.5	4.3 ± 1.1	3.9 ± 1.3
TEC Overall score (range = 0–9)	3.8 ± 2.0	4.7 ± 1.9	5.7 ± 1.8	5.8 ± 1.4	4.5 ± 1.9	5.2 ± 2.0
**Noticing Details Composite**	−0.1 ± 3.2	−0.7 ± 2.8	0.5 ± 2.2	1.3 ± 2.3	−0.1 ± 2.7	0.5 ± 2.4
TVPS—DIS scaled score	9.4 ± 3.5	9.2 ± 3.4	9.3 ± 2.3	10.1 ± 3.2	8.4 3.6	9.8 ± 3.2
TVPS—SPA scaled score	9.5 ± 4.9	10.6 ± 4.2	9.4 ± 4.0	12.9 ± 3.5	9.0 ± 3.6	12.0 ± 4.2
TVPS—FGR scaled score	10.6 ± 4.0	10.5 ± 3.1	11.5 ± 3.9	12.5 ± 2.6	11.5 ± 3.3	12.0 ± 2.6
Embedded figures (correct of 13)	5.4 ± 2.8	6.3 ± 3.8	6.5 ± 2.8	8.2 ± 3.0	5.6 ± 2.7	6.9 ± 3.5
**Impulse Control Composite**	−0.2 ± 2.9	−0.5 ± 2.4	0.8 ± 2.3	0.7 ± 2.2	−0.1 ± 1.8	0.2 ± 3.8
Simon Says (correct of 9)	5.6 ± 3.0	5.5 ± 3.3	4.0 ± 2.6	3.1 ± 3.5	4.2 ± 2.6	3.9 ± 3.4
Walk a line (s)	10.2 ± 11.2	10.5 ± 15.9	16.1 ± 13.0	14.7 ± 9.3	8.7 ± 8.4	13.9 ± 15.4
Draw a circle (s)	15.2 ± 18.3	13.9 ± 14.5	13.5 ± 10.7	15.4 ± 11.6	13.8 ± 14.9	16.0 ± 23.9
Long speech interrupt (1/0)	5/28	5/24	1/10	4/8	2/18	2/18
Prize bin (s)	78.4 ± 73.4	71.4 ± 55.0	80.7 ± 68.9	72.8 ± 46.1	60.0 ± 33.5	78.7 ± 43.3

Broader categories are bolded. IV = intervention; TEC = Test of Emotion Comprehension; TVPS = Test of Visual Perceptual Skills; DIS = discrimination; SPA = spatial relationships; FGR = visual figure ground; NA = Not applicable.

**Table 5:** Changes in scores (Post-Pre) and the adjusted intervention effect (ITT analysis).

Variables	Transportation Safety Group ^a^ (*n* = 30)	Dog Safety Group ^a^ (*n* = 34)	Adjusted Effect ^a,b^	*p*-Value ^b^
**Dog Knowledge and Behavior**				
Knowledge quiz	−0.32 ± 1.81	0.17 ± 1.93	0.51 ± 0.42	0.230
	(−0.99, 0.34)	(−0.49, 0.84)	(−0.32, 1.33)	
Dog photos	0.33 ± 2.17	0.24 ± 2.27	0.32 ± 0.52	0.448
	(−0.48, 1.14)	(−0.56, 1.03)	(−0.68, 1.34)	
Dollhouse safe behavior	0.54 ± 1.55	0.48 ± 1.75	−0.02 ± 0.39	0.970
	(−0.06, 1.13)	(−0.15, 1.11)	(−0.79, 0.76)	
Live dog risky behavior ^c^	0.09 ± 0.76	−0.18 ± 0.58	−0.38 ± 0.19	0.057
	(−0.28, 0.45)	(−0.43, 0.07)	(−0.75, 0.00)	
**Perspective Taking Composite**	0.36 ± 2.62	0.14 ± 2.45	0.49 ± 0.53	0.357
	(−0.64, 1.36)	(−0.73, 1.01)	(−0.55, 1.53)	
**Noticing Details Composite**	−0.28 ± 2.35	0.73 ± 2.10	1.20 ± 0.51	0.021
	(−1.23, 0.67)	(−0.03, 1.48)	(0.21, 2.20)	
**Impulse Control Composite**	−0.57 ± 2.01	0.11 ± 2.44	0.70 ± 0.63	0.274
	(−1.46, 0.32)	(−0.92, 1.14)	(−0.55, 1.95)	

^a^ Mean ± SD, and 95% CI. ^b^ Adjusted for age, gender and pre-scores in a General Linear Regression Model; ^c^ Post scores. Broader categories are bolded.

**Table 6:** Changes in scores (Post-Pre) and the adjusted intervention effect (per protocol, Level ≥ 9).

Variables	Transportation Safety Group ^a^ (*n* = 30)	Dog Safety Group: Compliant ^a^ (*n* = 12)	Adjusted Effect ^a,b^	*p*-Value ^b^
**Dog Knowledge and Behavior**				
Knowledge quiz	−0.32 ± 1.81	0.50 ± 1.17	1.05 ± 0.50	0.037
	(−0.99, 0.34)	(−0.24, 1.24)	(0.06, 2.03)	
Dog photos	0.33 ± 2.17	1.00 ± 1.76	1.21 ± 0.61	0.046
	(−0.48, 1.14)	(−0.12, 2.11)	(0.02, 2.40)	
Dollhouse safe behavior	0.54 ± 1.55	0.96 ± 1.59	0.15 ± 0.54	0.779
	(−0.06, 1.13)	(−0.05, 1.97)	(−0.90, 1.20)	
Live dog risky behavior ^c^	0.09 ± 0.76	−0.20 ± 0.50	−0.43 ± 0.25	0.090
	(−0.28, 0.45)	(−0.62, 0.22)	(−0.91, 0.05)	
**Perspective Taking Composite**	0.36 ± 2.62	−0.14 ± 2.24	0.74 ± 0.73	0.317
	(−0.64, 1.36)	(−1.56, 1.29)	(−0.70, 2.17)	
**Noticing Details Composite**	−0.28 ± 2.35	1.01 ± 1.80	1.58 ± 0.70	0.024
	(−1.23, 0.67)	(−0.20, 2.22)	(0.20, 2.96)	
**Impulse Control Composite**	−0.57 ± 2.01	−0.15 ± 2.00	0.83 ± 0.65	0.199
	(−1.46, 0.32)	(−1.82, 1.52)	(−0.44, 2.09)	

^a^ Mean ± SD, and 95% CI. ^b^ Adjusted for age, gender and pre-scores in a GLM; ^c^ Post scores. Broader categories are bolded.

**Table 7:** Descriptive data and model results for post-intervention and change.

Variables	Transportation Safety Group ^a^ (*n* = 30)		Dog Safety Group: Compliant ^a^ (*n* = 12)		Dog Safety Group: Non-Compliant ^a^ (*n* = 22)		Compliant vs. Transportation ^a^	Non-Compliant vs. Transportation ^a^	Compliant vs. Non-Compliant ^a^
	Post	Change	Post	Change	Post	Change	*p* ^b^, *adjusted effect* ^a,b^	*p* ^b^, *adjusted effect* ^a,b^	*p* ^b^, *adjusted effect* ^a,b^
**Dog Knowledge and Behavior**									
Knowledge quiz	5.3 ± 1.7	−0.32 ± 1.81	6.5 ± 1.3	0.50 ± 1.17	5.4 ± 1.8	0.00 ± 2.14	0.037	0.731	0.086
	(4.6, 5.9)	(−0.99, 0.34)	(5.7, 7.3)	(−0.24, 1.24)	(4.6, 6.2)	(−0.97, 0.97)	1.05 ± 0.50 (0.06, 2.03)	0.16 ± 0.47 (−0.76, 1.09)	1.01 ± 0.58 (−0.12, 2.14)
Dog photos	5.1 ± 2.3	0.33 ± 2.17	6.7 ± 1.2	1.00 ± 1.76	5.4 ± 2.6	−0.18 ± 2.44	0.046	0.848	0.028
	(4.2, 6.0)	(−0.48, 1.14)	(5.9, 7.4)	(−0.12, 2.11)	(4.2, 6.6)	(−1.26, 0.90)	1.21 ± 0.61 (0.02, 2.40)	−0.11 ± 0.57 (−1.22, 1.00)	1.44 ± 0.63 (0.20, 2.68)
Dollhouse safe behavior	3.6 ± 2.1	0.54 ± 1.55	3.0 ± 2.0	0.96 ± 1.59	3.9 ± 1.8	0.20 ± 1.82	0.779	0.918	0.850
	(2.8, 4.3)	(−0.06, 1.13)	(1.8, 4.3)	(−0.05, 1.97)	(3.0, 4.7)	(−0.65, 1.05)	0.15 ± 0.54 (−0.90, 1.20)	−0.05 ± 0.44 (−0.92, 0.82)	0.12 ± 0.63 (−1.11, 1.35)
Live dog risky behavior	0.09 ± 0.76	NA	−0.20 ± 0.50	NA	−0.17 ± 0.63	NA	0.090	0.064	0.649
	(−0.28, 0.45)		(−0.62, 0.22)		(−0.52, 0.18)		−0.43 ± 0.25 (−0.91, 0.05)	−0.43 ± 0.22 (−0.87, 0.01)	0.11 ± 0.24 (−0.36, 0.58)
**Perspective Taking Composite**	−0.2 ± 3.0	0.36 ± 2.62	1.0 ± 1.7	−0.14 ± 2.24	0.6 ± 2.4	0.29 ± 2.61	0.317	0.497	0.842
	(−1.4, 0.9)	(−0.64, 1.36)	(−0.1, 2.1)	(−1.56, 1.29)	(−0.4, 1.7)	(−0.89, 1.48)	0.74 ± 0.73 (−0.70, 2.17)	0.42 ± 0.62 (−0.80, 1.65)	0.12 ± 0.61 (−1.07, 1.31)
**Noticing Details Composite**	−0.7 ± 2.8	−0.28 ± 2.35	1.3 ± 2.3	1.01 ± 1.80	0.5 ± 2.4	0.58 ± 2.27	0.024	0.070	0.454
	(−1.8, 0.4)	(−1.23, 0.67)	(−0.2, 2.9)	(−0.20, 2.22)	(−0.6, 1.5)	(−0.45, 1.61)	1.58 ± 0.70 (0.20, 2.96)	1.03 ± 0.57 (−0.09, 2.16)	0.50 ± 0.67 (−0.81, 1.81)
**Impulse Control Composite**	−0.5 ± 2.4	−0.57 ± 2.01	0.7 ± 2.2	−0.15 ± 2.00	0.2 ± 3.8	0.24 ± 2.68	0.199	0.352	0.403
	(−1.5, 0.5)	(−1.46, 0.32)	(−0.8, 2.3)	(−1.82, 1.52)	(−1.5, 2.0)	(−1.18, 1.67)	0.83 ± 0.65 (−0.44, 2.09)	0.66 ± 0.71 (−0.73, 2.04)	−0.89 ± 1.06 (−2.97, 1.18)

^a^ Mean ± SD, and 95% CI. ^b^ Comparing change after adjusting for age, gender and pre-scores in a GLM analysis, except live dog risky behavior which compares post score adjusting for age and gender. Broader categories are bolded.

**Table 8:** Descriptive post-intervention data: Percentage of children and parents in each group.

	Dog Safety	Dog Safety	Transportation
	Compliant	Non-Compliant	Safety
**Child Report**			
Enjoy playing website games? (% yes)	90	94	89
Enjoy watching website videos? (% yes)	73	89	52
Other kids would enjoy website? (% yes)	64	50	70
**Parent Report**			
Child enjoyed website? (% “a lot”)	92	60	68
You enjoyed website? (% “a lot”)	58	20	19
Will continue using website at home (% yes)	72	45	45
Would recommend website to others (% yes)	82	69	54
